# Improving biomass and carbohydrate production of microalgae in the rotating cultivation system on natural carriers

**DOI:** 10.1186/s13568-023-01548-5

**Published:** 2023-04-29

**Authors:** Zahra Mousavian, Maliheh Safavi, Alireza Salehirad, Farzaneh Azizmohseni, Mahnaz Hadizadeh, Saeed Mirdamadi

**Affiliations:** 1 Department of Biotechnology, Iranian Research Organization for Science and Technology (IROST), P. O. Box 3353-5111, Tehran, Iran; 2 Department of Chemical Technologies, Iranian Research Organization for Science and Technology (IROST), P. O. Box 3353-5111, Tehran, Iran; 3 Iranian Research Organization for Science and Technology (IROST), Sh. Ehsani Rad St., Enqelab St., Parsa Sq., Ahmadabad Mostoufi Rd., Azadegan Highway, P. O. Box 3353-5111, Tehran, 3353136846 Iran

**Keywords:** Attached microalgae cultivation, Biomass harvesting, Natural carrier, Suspension culture, Microalgae exopolysaccharide

## Abstract

Biofilm-based algal technologies have gained popularity due to higher biomass productivity, efficient harvesting, and water-saving over suspended growth systems. A rotating attached system was designed to assess the biofilm-forming capacity of different isolated microalgal strains from the Persian Gulf. Four microalgal strains, including two *Chlorella* sp., one *Picochlorum* sp. and one filamentous cyanobacterium *Desmonostoc* sp. were cultivated on four carriers: jute, cotton, yarn and nylon. The carriers’ physicochemical surface characteristics and attachment effects, like contact angle, were investigated. The incorporated biomass and exopolysaccharides (EPS) content in the suspended and biofilm system was calculated and compared. The results showed that the cyanobacterium strain had the biofilm formation capability on both jute and cotton in the attached cultivation system. Under the same culture conditions, the biomass productivity on jute and cotton carriers was significantly higher (4.76 and 3.61 g m^− 2^ respectively) than the growth in aqueous suspension (1.19 g m^− 2^ d^− 1^). The greatest incorporated exopolysaccharides amount was observed on jute (43.62 ± 4.47%) and the lowest amount was obtained from the growth on positive charge yarn (18.62 ± 1.88%). This study showed that in comparison with planktonic growth, the colonization of cyanobacterial cells and subsequent production of extracellular matrix and biofilm formation can lead to increased biomass production.

## Keypoints

• Biofilm algal cultivation provides easy biomass harvesting by mechanical scraping

• The physio-chemical properties of the carrier influenced the attachment of microalgae cells

• The filamentous strain showed optimal attachment performance on jute and cotton respectively

## Introduction

In the last two decades, microalgae have gained increasing research attention as a valuable source of bioproducts for a variety of commercial biotechnological applications, including human and animal nutrition, cosmetics, and pharmaceutical industries (Barros et al. 2018; Borowitzka 2013; Miguel et al. 2021). Microalgal cultivation is predominantly done under photoautotrophic conditions, either in raceway ponds or closed photobioreactors (PBRs)(Borowitzka 2013; Rincon et al. 2017). In suspension cultivation such as open ponds/raceways, the dry biomass concentration g (DW) is typically around 0.5 g(DW) L ^− 1^, but in enclosed systems, the production rate has been improved to 2‒10 g(DW) L^-1^(Gross et al. 2015). Although PBRs produce more biomass, they also have some drawbacks, such as limited light utilization, high production costs, and difficulties in scale-up (Wang et al. 2014; Melo et al. 2017). Developing low-cost processes and improving biomass productivity is critical because of the significant challenges in microalgae suspension culture, such as high water demand, high harvesting and operating costs, and low biomass yield (Berner et al. 2014; Gross and Wen 2014a; Mantzorou and Ververidis 2019). Significant research is concentrated on both technological and ecological aspects of the cultivation system to optimize large-scale microalgae production to maximize growth rate while being economically sustainable and environmentally friendly (Johnson and Wen 2010; Christenson and Sims 2012; Karimi et al. 2021). Biofilm cultivation is an innovative and appealing microalgae cultivation technology offering the potential to lower water and energy requirements (Ozkan et al. 2012).

The first rotating algal biofilm with a polystyrene disk design was used in the 1980s for municipal wastewater treatment (Przytocka-jusiak et al. 1984). Recent years have seen increasing interest in algal biofilm reactors developed. Currently, the design of algal biofilm bioreactors is mostly focused on different geometrical configurations and different support or attachment materials. Some examples of contemporary biofilm systems in microalgae cultivation for diverse purposes include flat rotating disks (Blanken et al. 2014), cylindrical rotating drums (Christenson and Sims 2012), and conveyor rotating belts (Gross et al., 2013; Wood et al. 2022). As compared to conventional harvesting methods including membrane filtration, centrifugation, and sedimentation, algal biofilm harvesting can overcome the high cost and energy consumption associated with it. This alternative growing technique has several benefits over suspended microalgae growth, including simpler gathering and drying, higher energy efficiency, lower cost, and denser microalgae biomass (Zhuang et al. 2020). Total algal harvesting in non-suspended systems, contains 10 to 20%, biomass (solid) content which is higher than that obtained by centrifugation recently and is considered a time-saving and energy-efficient potential method. In the biofilm system, harvestable dry-weight biomass yield is reported 100–200 g (DW) kg^− 1^ and it can reach up to 208 g kg^− 1^ dry weight ( Sebestyén et al. 2016). Reduced costs associated with algae harvesting as a result of the biomass being easily scraped from the surface; the lack of a requirement for arable land; and enhanced biomass yield are all advantages of employing this alternative cultivation strategy (Blanken et al. 2014; Gross and Wen 2014a). The performance of the overall production culture system depends significantly on the substrate on which microalgae attach (Assis De Rodrigues et al. 2019). Microalgae cells should be strongly immobilized during the growth phase and easily released during harvesting on an appropriate support carrier (Zhao et al. 2021). The algae-material attachment has been explained by various hypotheses including, acid-base interactions (Ozkan and Berberoglu 2013a), hydrophobic interactions (Klein et al. 2014), and surface energy (Finlay et al. 2002). Initiation of algal colonization is significantly influenced by physicochemical material factors including surface tension, water contact angle, wettability, hydrophobicity, and surface roughness, as well as the microalgae strain (Tsavatopoulou and Manariotis 2020). In general, materials have been suggested to increase microalgae biofilm development that are cost-effective, durable (resistant to degradation), and commercially viable (Assis De Rodrigues et al. 2019). The effectiveness of various materials, such as natural and synthetic substrates, in the creation of algal biofilms, has been the subject of much research (Christenson and Sims 2012; Genin et al. 2014; Venable and Podbielski 2019). It has been demonstrated that cotton (Wood et al. 2022; Christenson and Sims 2012; Gross and Wen 2014b), nylon mesh (Lee et al. 2014), and polystyrene foam (Johnson and Wen 2010) have been proven best performers in terms of cell binding, durability and cost of the support material.

The high efficiency in the performance of the non-attachment microalgae culture system was mainly attributed to its extracellular polymeric substance (EPS)matrix. EPS is a polymeric network consisting of carbohydrates, proteins, lipids, and other metabolites that play a critical role in cell attachment and the production of microalgae biofilms (Pippo et al. 2013; Carbone et al. 2017). Exopolysaccharides comprise a dominant part of the EPS and represent a key role in the attachment of cell microalgae to surfaces (Pippo et al. 2013). The exopolysaccharides facilitate cell adhesion to substrates by binding cells together and promoting embedding in the biofilm matrix. EPS serves to sustain the growing biofilm on the surface and increase its thickness during the development phase as EPS supports cell-cell communication (Cheah et al. 2021).

This investigation focuses on the ability of three microalgae and one cyanobacterium strain, both newly isolated from Iran’s Persian Gulf and Oman Sea in Iran, to produce biofilms. A rotating attached algal bioreactor with different substrates was used to achieve high algal productivity and cost-effective harvesting processes. Also, the biomass production and carbohydrate content of microalgae in suspended culture and biofilm systems were compared.

## Materials and methods

### Microalgal culture

The strains of *Chlorella sorokiniana*, *Chlorella* sp., *Picochlorum* sp. and *Desmonostoc* sp. used in this study were isolated from the Persian Gulf and the Qeshm Island (26°32 N, 53°56 E), at the southern of Iran and deposited in Persian Type Culture Collection (PTCC) with accession numbers M8011, M8010, M6032 and 1968 respectively. Algae strains were grown in Bold’s basal medium (BBM) using an inoculum of 1:10 in bath culture. BBM stock solutions (per liter) contain 2.5 g calcium chloride dehydrate, (CaCl_2_.2H_2_O); 25 g sodium nitrate (NaNO_3_); 7.5 g potassium phosphate (K_2_HPO_4_); 7.5 g magnesium sulfate heptahydrate (MgSO_4_.7H_2_O); 2.5 g sodium chloride (NaCl); 11.42 g boric acid (H_3_BO_3_); 17.5 g monopotassium phosphate (KH_2_PO_4_); alkaline EDTA solution, acidified iron solution; trace metal solution containing 1.44 g manganese (II) chloride tetrahydrate (MnCl_2_.4H_2_O); 8.82 g zinc sulfate heptahydrate (ZnSO_4_.7H_2_O); 1.57 g copper (II) sulfate pentahydrate (CuSO_4_.5H_2_O); 0.71 g molybdenum trioxide (MoO_3_); and 0.49 g cobalt (II) nitrate hexahydrate (Co(NO_3_)2.6H_2_O)(Bold 1949). The strains were cultivated at room temperature, 100–150 rpm agitation (Stuart Scientific, model 5STR8), continuous illumination using cold light fluorescent (FLUORA, L36W/77, OSRAM) in the culture and photoperiod 12 L:12D. Biofilm culture was inoculated from suspended culture and was grown in the same suspension condition (Melo et al. 2017).

### Adhesion and biofilm formation tests

#### Preliminary substrate adhesion tests

**Fig. 1:**
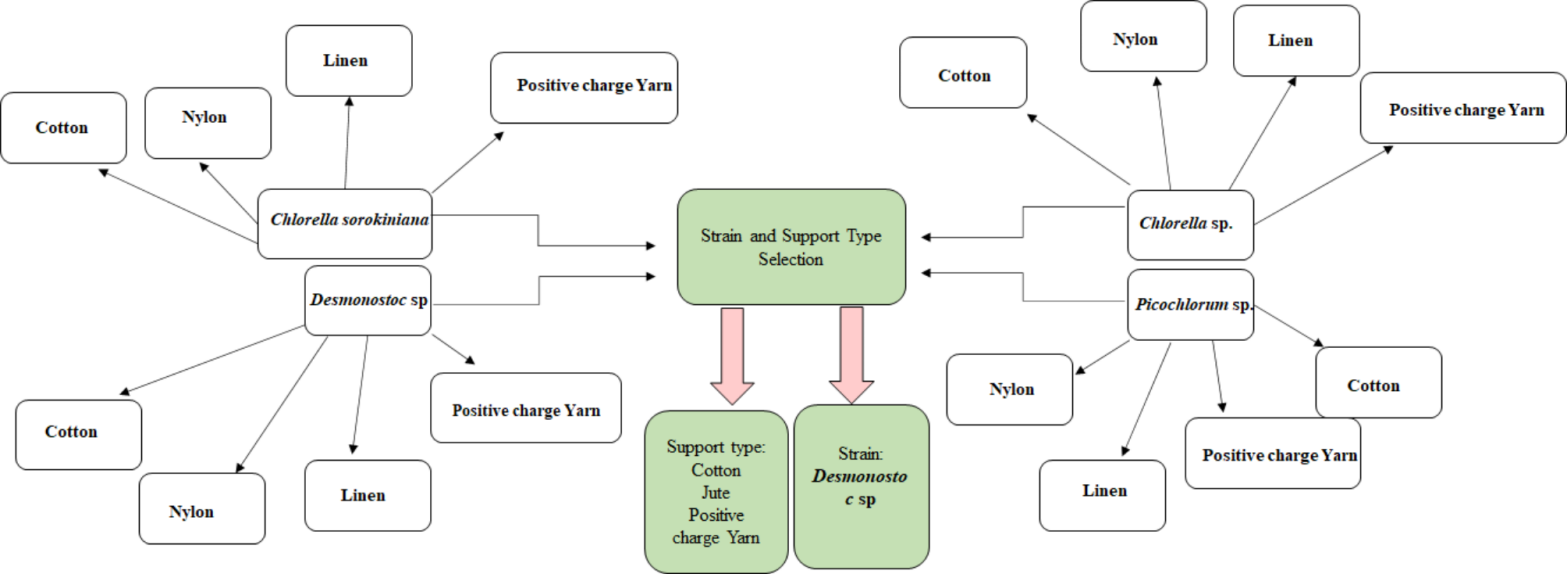
Experimental design for selecting the appropriate substrate and microalgae/ cyanobacteria strains for the attached-based culture system

The materials selected for the experiments of adhesion potential of microalgae/ cyanobacteria strains included nylon, cotton, jute, linen, and positive-charged yarn. Material choice was based on some properties such as non-toxicity, availability, simplicity of manipulation, and reported hydrophilic/hydrophobic behavior in previous studies (Sekar et al. 2004; Irving and Allen 2011). In the first part of this study, all the materials were cut into 2 cm × 2 cm coupons. These carriers were autoclaved at 121 °C for 20 min before being placed on a 9 cm diameter glass petri dish containing 30 mL of sterile BBM and inoculated with exponential phase microalgae cells as inoculum (Melo et al. 2017). The surface elements of four algal species (three unicellular strains (*Chlorella sorokiniana*, *Chlorella* sp., *Picochlorum* sp.) and one filamentous cyanobacterium strain (*Desmonostoc* sp.) were evaluated independently (Fig. 1). The plates were placed in triplicate for each test in an orbital shaker (GFL 3005, Germany) with constant agitation of 160 rpm. The culture conditions for Petri dishes are held at the same temperature, light and rotation as previously described for the suspension cultures. A petri dish without a carrier as control was placed under the same culture condition. The carrier adhesion monitoring of microalgae/ cyanobacteria strains was performed through a photographic recording and dry weight measurement of each carrier (Carbone et al. 2017).

### Laboratory-scale microalgae culture

#### Microalgae culture in rotating photobioreactor

**Fig. 2:**
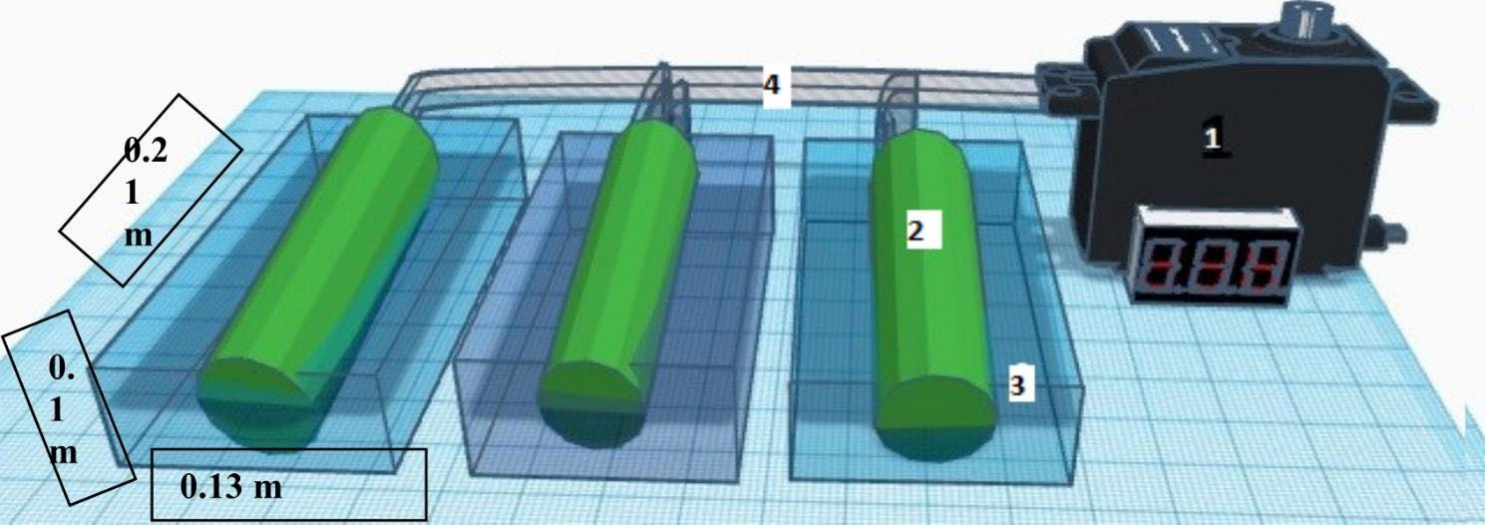
Scheme of rotating biofilm photobioreactor. This device consists of (1) a motor. (2) Floating cylinders in culture medium (3) culture chamber containing the culture medium. (4)conveyor. The vessel size is 0.21 × 0.13 × 0.1 m^3^. 3 rotating cylinders horizontally are rotated in BBM medium each time. The chamber was 24 h continuously irradiated by two fluorescent lamps

The setting for the experiment included three chamber, three cylinders, motor, eight lamps, and conveyor (Fig. 2). Using the innovative rotating biofilm culture concept, a novel bioreactor was designed to perform biomass production of selected algae and cyanobacterium strains as a biofilm. Instead of using a rotating algal biofilm reactor like the Algal disk (Blanken et al. 2014), the RABR (Christenson and Sims 2012), or the PRBC reactors, we employed three Plexiglas containers (21 cm 13 cm 10 cm, L W H) placed in a shaft cylinder rotating at 2.8 rpm. The growth surface, with the rotation of the cylindrical, was exposed to the medium and the air alternately.

The experiments were conducted in three chambers using jute, cotton and positive-charged yarn as support materials for selected cyanobacterium strain *Desmonostoc* sp. which has been chosen from previous test “Preliminary substrate adhesion tests”. Each chamber was inoculated with 400mL of effluent from the suspension culture as seeding media. Throughout the experiment, 12:12 L:D fluorescent light cycles (FLUORA, L36W/77, OSRAM) cycle of 12:12 L:D were utilized.

Before being filled with media, each container was washed with water, disinfected with ethanol 70%(v/v), rinsed with sterilized BBM medium, and then filled with inoculum media. Following scrapping the cylinders, the reactors were replaced with BBM medium, and regeneration was carried out using microalgae cells that had remained attached to the substance that had covered the cylinders as inoculum.

The formation and development of the biofilm were monitored visually by photographic recording and dry weight measurement. Biofilm dry weight was measured by drying biomass scraped from cylinders at 60 °C overnight. Growth measurements in every chamber were performed three times.

#### Suspension culture

Suspended cultures were grown under the same growth condition including 70 mol photons m^− 2^s^− 1^ irradiance, BBM media, and room temperature as the biofilm cultures. The suspended culture was operated with a 12/12 h dark/light cycle in the same way as the biofilm culture in the carrier test of the **microalgae culture in rotating photobioreactor** section. Biomass monitoring was carried out with the harvesting of suspended biomass and concentrated using a centrifuge at 8,000 g for 10 min (SIGMA, High-Speed Refrigerated Centrifuge, 3-30KS)(Christenson and Sims 2012).

### Evaluation of material surface physico-chemical properties

Contact angle measurements were used to assess the physicochemical parameters, including calculating the surface energy of the algae cells and the carriers. The contact angle was determined using the sessile drop tests. Briefly, 50µL of the distilled water and glycerol as reference liquids was pipetted onto the surface of the material with and without microalgae cells, and the contact angles of all the probe liquids were recorded using a goniometer (Model CAG-10, Jikan company, Iran). The contact angle for each drop is the average of the angles measured on the left and right sides of the drop.

In general, the calculation of the free energy surface based on measurements of the contact angle of liquids deposited on the solid surface is used in Zisman’s method. The surface energies were calculated by the contact angles using Zisman’s equation, i.e.,

1 $$cos\theta = 1 + b\left( {\gamma c - \gamma 1} \right)$$

where γ_L_ is the surface energy of the liquid, θ is the contact angle, and is the tangent of an angle between the x-axis and the straight line, is the approximation of the measurement results, and γc is the critical surface free energy that differs from the quantity γ_s_. The relationship between γc and γs is described as follows: (Żenkiewicz 2007).

2 $$\gamma s = \left( {b\gamma c + 1} \right)2/\left( {4b} \right)$$

### Scanning electron microscopy and infrared spectroscopic analysis of biofilm carriers

FESEM was used to visualize and compare the biofilm formation of filamentous strain *Desmonostoc* sp. on various carriers. The FESEM sample was prepared following the method of Mizan et al. (2018). After fixation of samples in 2% glutaraldehyde in PBS at room temperature for 4 h, the fixed samples were stepwise treated with an ethanol gradient (50, 60, 70, 80, 90, and 100% for 15 min). Successively dehydrated samples were treated by soaking in hexamethyldisilizane in ethanol (33, 50, 66, and 100%,15 min). Using an electron microscope TESCAN MIRA SEM (Czech Republic) equipped with a 15 kV acceleration voltage and a 15 mm working distance, we observed the gold-palladium sputter-coated samples (Mizan et al. 2018).

The technique of Fourier transform infrared spectroscopy (FTIR) has been recommended for the study of macromolecules and the surface colonization of microorganisms (Khoironi et al. 2019). The IR spectra of the microalgae/ cyanobacterium biofilm carrier samples were recorded on a Bruker Tensor 27 FTIR spectrometer between 400 and 4000 cm^− 1^ (Baky et al. 2013).

### Exopolysaccharide extraction

Dried microalgae biomass (1 gr) was resuspended in acid hydrochloric 0.07% (ratio 1:10). The mixture was incubated in a shaking water bath at 90 °C for 4 h. The suspension was centrifuged for 10 min at 10,000×g at 4 °C and 96% (v/v) of cold ethyl alcohol was added to the supernatant at a ratio of 2:1. After overnight incubation at 4 °C with constant mechanical stirring, the mixture was centrifuged at 10,000×g for 10 min at 4 °C. Finally, the PS in dry form was obtained by lyophilization of the precipitate (Ale et al. 2012).

### Calculations and statistical analysis

Biomass production was calculated based on the gravimetric method (dry weight-based). Analysis of variance (ANOVA) with a confidence level of P < 0.05 was used to compare biomass productivity among different cultivation systems, growth substrates, and EPS content. According to Shen et al., photobioreactor performance was measured as surface biomass yield (g m^− 2^), and surface adhesion biomass productivity (PS, g m^− 2^ day^− 1^, Eq. 3), which is based on the surface area of the substrate.

3 $$PS = \frac{{DW2 \times V2}}{{A \times CP}}$$

where DW_2_ is the biomass dry weight (g L^− 1^) and V_2_, A and CP are the volume (l) of the re-suspended medium, the area (0.0238 m^2^) of the substrate material and the culture period.

## Result

### Preliminary substrate adhesion

**Fig. 3:**
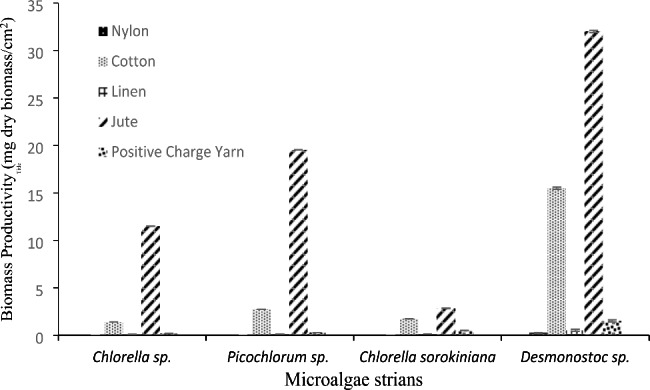
Comparison biomass productivity (mg dry biomass cm^-2^) between different microalgae strains on various supporting materials (nylon, cotton, linen, jute and positive charge yarn) (Value presented as mean ± SD)

During the initial stages of substrate colonization, five different attaching materials, including nylon, cotton, jute, linen, and positive charge yarn, were evaluated to determine the impact of surface roughness on algal attachment. Figure 3. exhibits a variety of attachment performances as dry weight (mg dry mass. cm^− 2^) which was the requirement for including these materials in this investigation.

### Microalgal biofilm growth kinetics

The *Desmonostoc* sp. strain was grown in semi-continuous culture on three carriers in a rotating photobioreactor for about 91 days. Microalgal biomass productivity of the rotating photobioreactor system was compared with that of the suspended system to evaluate the advantage of microalgae/ cyanobacteria cultivation. Following the laboratory-scale testing, the materials exhibiting the best attachment (mesh jute, cotton and positive charge yarn with various surface textures) were further tested on a rotating system to evaluate long-term cell-attached growth as a function of different materials. Cells were incubated on a rotating biofilm system for the first 27 days for initial attachment and followed by 5 cycles of repeated harvesting and re-growing at 7–22 days/cycle for a total of 3 months of attached growth.

**Fig. 4:**
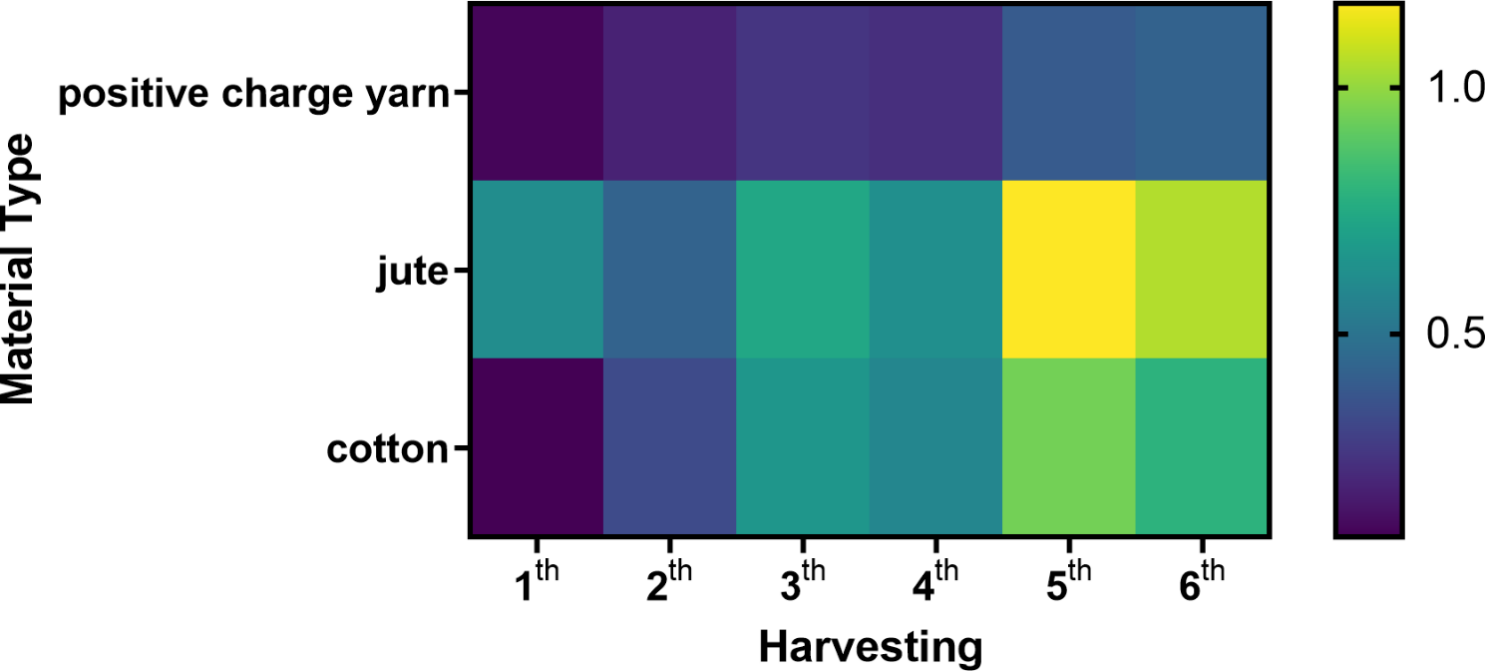
Heat map of biomass productivity (g m^-2^) of *Desmonostoc* sp. strain in harvesting steps

As shown in Fig. 4, during the initial harvesting there was a significant difference in the thickness of the biofilm on positive charge yarn and cotton surfaces and higher attachment occurred on jute. During additional harvesting steps, jute and cotton had approximately the same biomass yield. Cotton, on the other hand, is more water-absorber than jute.

**Fig. 5:**
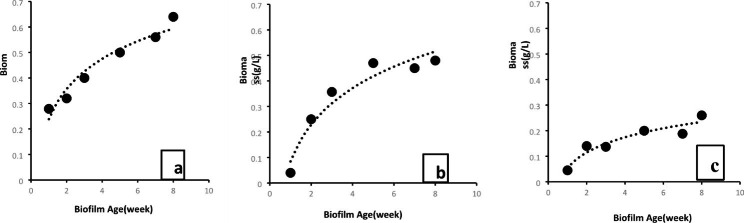
Growth kinetics curves for cyanobacterial biofilms grown on three different materials: jute (**a**), cotton (**b**) and positive charge yarn (**c**)

The growth curves of total biomass in each support material in the biofilms are illustrated in Fig. 5. In the jute, cotton and positive charge yarn supports (Fig. 5a, b and c respectively), the first-order kinetics were adjusted to the biomass growth curve (R^2^ = 0.938, R^2^ = 0.933 and R^2^ = 0.894 respectively).

The cotton support had a maximum peak of 0.47 g L^− 1^ of total biomass during the 68th day of operation (Fig. 5b), yielding a productivity of 0.014 g d^− 1^. The jute support presented a maximum production and productivity peak of 0.64 g L^− 1^ occurred on the 91st day of cultivation (Fig. 5a). As shown in Fig. 5a, at the 13th week of cultivation, the curve of the jute carrier material was still growing, indicating that its production remained increasing exponentially throughout the biomass’s growing time. In this support, the logarithmic phases of the cell growth curve persisted. The positive charge yarn support achieved a maximum total biomass production of 0.3 g L^− 1^ in 68 days (Fig. 5c).

### Evaluation of material surface physico-chemical properties on algal attachment

#### Contact angles and surface properties

The surface physico-chemical properties, including water and glycerol contact angles, as well as the critical surface free energy and free surface energy of the carrier, were evaluated. As shown in Table 1, the liquid contact angles were very different between raw materials and materials with microalgae cells on the surface.

**Table 1:** Water contact angle, glycerol contact angle, critical surface free energy and surface free energy of the surface the jute, cotton and positive charge yarn such as water contact angle, glycerol contact angle, critical surface free energy and surface free energy as raw and coated with microalgae cell

Carriers	Θ _Water_	Θ _Glycerol_	Equation	γ_C_	γ_S_	Biomass production (g/ m^2^)
Jute Raw	55.78	52.30	y = -0.0057x + 0.9742	150	150.92	-
Jute covered with Algae	81.29	70.81	y = -0.0206x + 1.6366	113.99	-136	66.74
Cotton Raw	0	70.83	y = 0.0781x − 4.6235	72.72	142.79	-
Cotton covered with Algae	48.42	68.78	y = -0.0347x + 2.8593	91.011	-124.49	44.41
positive charge yarn Raw	0	98.11	y = 0.1327x − 8.5526	72.79	214.09	-
positive charge yarn covered with Algae	64.39	76.90	y = 0.0289x − 1.6466	72.66	83.04	21.54

According to the Young–Dupre theory, hydrophobicity (low wettability) and hydrophilicity (high wettability) are realized when the contact angle of the water droplet is close to 90° or 0°, respectively (Licari and Swanson 2011).So, cotton with θ = 0 and jute with θ > 90 were strongly hydrophilic and hydrophobic surfaces. The total amount of microalgae deposited on each surface corresponded to 66.74, 44.41 and 21.54 g m^− 2^ for jute, cotton, and positive charge yarn. This result indicated a strong relationship between water contact angle and surface energy. When cyanobacterium cells were attached to surfaces, their wettability was changed. So, the two carriers’ cotton and yarn became hydrophobic with increasing contact angle, but jute hydrophobicity adjusted with microalgae cell attachment (Table 1).

### Field emission scanning electron microscopy of microalgae biofilm- growing

**Fig. 6:**
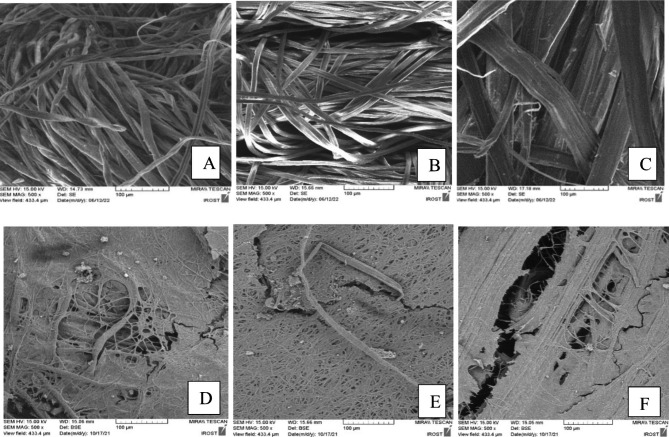
FESEM micrographs of raw carrier and algal attachment on cotton, yarn and jute. (**A**) raw cotton (**B**) raw yarn. (**C**) raw jute. (**D**) cotton covered by cyanobacterium cells. (**E**) yarn covered by cyanobacterium cells and, (**F**) jute covered by cyanobacterium cells. The experiments were tested under rotating conditions

After quantitatively measuring cell adhesion, the effect of surface texture on cyanobacterium cell attachment under rotating conditions was evaluated using field emission scanning electron microscopy (FESEM) observation. The FESEM observations shown in Fig. 6 were in agreement with the quantitative cell attachment results shown in Table 1.

### Characterization by ATR-FTIR spectroscopy

**Fig. 7:**
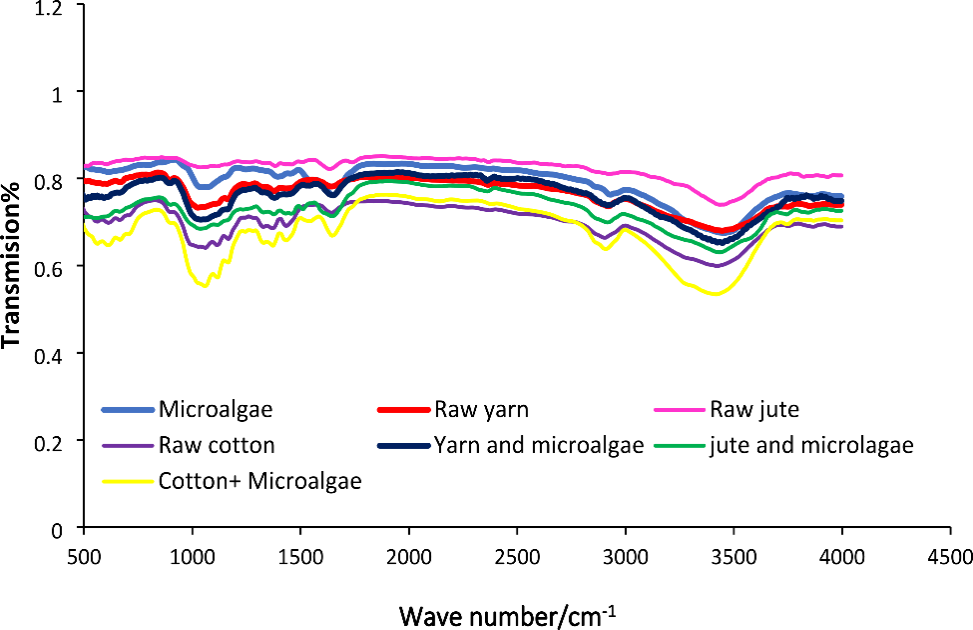
ATR-FTIR spectra of *Desmonostoc* sp., raw yarn, jute and cotton, yarn, jute and cotton covered by microalgae

Physical-chemical characterization of the cyanobacterium and the three raw and covered with cyanobacterium selected carriers (positive charge yarn, jute, and cotton) was performed afterwards by ATR-FTIR. FTIR spectra can be used to characterize and compare functional groups on the carrier surface that could form molecular interactions with those on the cell surface. All the spectra presented in Fig. 7, share an approximal similar shape pattern in the all-region. As shown in Fig. 7, overlapping bands have shown a similar pattern in the fingerprinting region of the FTIR spectrum, which can be attributed to the various functional group’s adsorption peaks in all samples. This may be explained by the fact that the functional groups did not play a role in the attaching process.

### Biomass production and carbohydrate content of attached and suspended systems

In the suspended culture, the biomass productivity was 1.19 g DW. m^− 2^ day^− 1^. Table 2. shows dry matter in terms of yield output and productivity of biomass associated with the biofilm recovered from the various materials. Jute produced the highest amount of biomass output and productivity (66.74 g m^− 2^, 4.76 g m^− 2^ d^− 1^). Despite the fact that the biomass yields from the two systems were measured in different units, the volume of liquid in each culture was the same, allowing for a comparison of the absolute quantity of biomass. On jute and cotton, biomass productivity was four and three times higher in biofilm culture than in suspended culture. More light penetration and increased surface area for biomass growth can be considered as the main reasons for the higher productivity of the biofilm culture system compared to suspended culture mode (Economou et al. 2015).

**Table 2:** Total algal biomass production, biomass productivity and EPS yield in the different carrier’s materials after 91 days (Value presented as mean ± SD)

Carrier Type	Total Biomass Production yield (g m^− 2^)	Total Biomass Productivity (g.m^− 2^ day^− 1^)	Polysaccharide Yield (%)
Positive charge yarn	21.54 ± 6.15	1.71 ± 0.79	18.62 ± 1.88
Jute	66.74 ± 8.28	4.76 ± 0.74	43.62 ± 4.47
Cotton	44.41 ± 7.38	3.61 ± 0.69	26.81 ± 4.25

The experimental EPS yield in suspended and immobilized cultivated filamentous strain *Desmonostoc* sp. was 23.78 ± 1.24%, and 43.62 ± 4.47% (in jute) respectively, but the positive charge yarn had the lowest amount.

The estimated total sugar content of suspended and various supporting materials using the analysis of variance suggested a statistically significant difference between culture type and sugar content (p-value < 0.05). Also, substantial differences in total sugar content have been identified between jute and other supports (p-value < 0.05).

The evidence presented in Table 2. suggests that the production of more EPS increased the cell attachment strength of cells to hydrophobic materials.

**Fig. 8:**
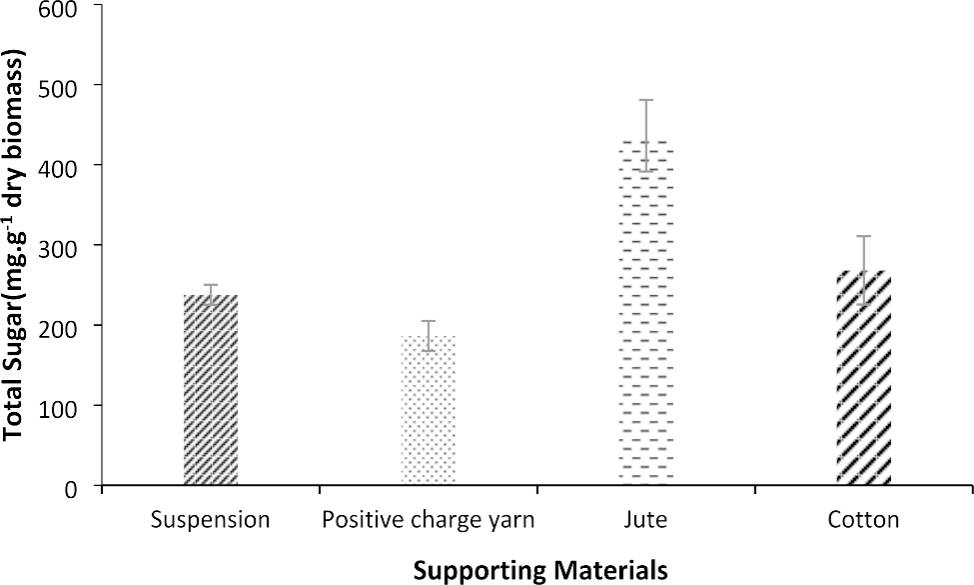
Carbohydrate content of suspended and attached systems on different carriers (jute, cotton, and positive charge yarn) (Value shown as mean ± SD).

Figure 8. confirmed the preferential attachment of filamentous cyanobacterium cells that occurred on jute and cotton due to EPS compared to flat surfaces (such as positive-charge yarn).

## Discussion

### Preliminary substrate adhesion

The result of the initial stages of substrate colonization indicated that microalgae were less likely to adhere to the nylon’s smooth surface than to other textured surfaces. Lower attachment of *Chlorella sorokiniana*, *Chlorella* sp. and *Picochlorum* sp. as unicellular strains were observed on all surfaces. *Desmonostoc* sp. *as* filamentous strain attached more strongly to jute, cotton and positive charge yarn in batch cultures. In general, the degree of filamentous strain biofilm formation was found to follow the sequence of carrier jute > linen > cotton > positive charge yarn > nylon. Our findings agree with the findings of Lee et al., who found that the architecture of microalgal biofilms is species-dependent, and attachment material selection is influenced by microalgal/ cyanobacteria species (Lee et al. 2014).

Additionally, the positive correlations between initial cell attachment and surface texture and roughness observed in this study are similar to those reported previously (Sekar et al. 2004; Renner and Weibel 2011; Achinas et al. 2019). Our results have been confirmed by the result of the Christenson and Sims study that showed the nylon rope did not achieve any harvestable growth (Christenson and Sims 2012). In this study, rough and textural surfaces were shown to be more effective for biofilm development, which contradicted the findings of Gross et al., who discovered that *Chlorella vulgaris* (UTEX #265) adhered better to nylon as a smooth surface (Gross & Wen, 2014). Also, Sweat and Johnson found that benthic diatoms attach better to a smoother substrate, such as smooth acrylic panels (Sweat and Johnson 2013).

In brief, we found that the size and shape variation of algae cells is affected by initial attachment on the surface (Achinas et al. 2019). The higher contact between the cell and surface when the cell is larger than surface features (as is the case for filamentous cells *Desmonostoc* sp.) has a significant impact on the adhesion trend compared to the interaction between the cell and surface when the cell is smaller than the surface features (as in *Chlorella sorokiniana, Chlorella* sp. and *Picochlorum* sp. cell cases). These findings were previously reported by Katainen et al. (Katainen et al. 2006).

Compared to the smooth surface of nylon, the texture and roughness of jute aid in capturing filamentous cells, exhibiting the highest amount of cell attachment. A similar phenomenon has been observed in algal-produced river biofilms (Zhang et al. 2019).

More chances for cell colonization on rough surfaces are probably due to having more acceptor sites and a larger surface area for cell attachment (Sousa et al. 2009). Microorganism cells on the rougher surface, on the other hand, are protected from fluid shear forces (Achinas et al. 2019).

Some researchers examined the various types of natural and synthetic carriers for algae biofilm cultivation (Christenson and Sims 2012; Gross 2015). The presence of polar-OH groups in the glucose unit of cotton as natural polysaccharides provides multiple hydrogens for binding to microalgae/ cyanobacteria exopolysaccharides. Recent studies on cotton as natural support have indicated that cotton has the best algal attachment performance (Gross 2015). Similar to this study result, Christenson & Sims confirmed cellulose-based support such as jute and cotton achieved greater cell adherence than polyester and acrylic (Christenson and Sims 2012). Some research suggests that the addition of cotton fabric is an appropriate support candidate for algal attachment, and it’s also more applicable for commercially feasible algal attachment (Johnson and Wen 2010; Gross et al. 2013a). According to findings from the first screening, the filamentous strain *Desmonostoc* sp. was chosen from the studied strains; jute, cotton, and yarn were selected for the next phases.

### Microalgal biofilm growth kinetics

To monitor microalgae growth, the biomass productivity and dry biomass yields of microalgae cultured on three biofilm system materials were evaluated. Our findings are in agreement with the fact that the initial attachment of algal cells to the fresh surface of the materials is crucial and can be time-intensive. Once the initial colonization occurs, the attachment of additional algal cells to the existing algal biofilm layer is relatively easy. This is also highlighted in Ozcan et al. study (Ozkan and Berberoglu 2013a).

The increasing biomass productivity from re-growing (2nd, 3rd and 4th harvesting) based on the hypotenuse of Schnurr et al. could be the result of lag phase elimination and symbiosis cells with each other due to the presence of residual EPS and biomass in previous growth respectively (Schnurr and Allen 2015).

Different surface properties of the substrates, such as roughness and hydrophobicity, have been reported to play roles in algal cell attachment (Fanesi et al. 2019). The influence roles of different surface properties of the substrates, such as roughness and hydrophobicity have been reported to play roles in algal cell attachment. Comparing the three carriers tested in this study, jute and cotton were the rougher materials, respectively, and, as a result, showed a higher adhesion of the cells in the same culture time. However, the roughness of the biofilms changes from rough to smothering over time based on the metabolic and nutritional transport rates of cells. An increase in the adhesion of microalgae can be explained by the use of meshed substratum as an alternative carrier material by increasing the surface roughness (Fanesi et al. 2019). The pore size of the cotton and jute mesh used in this study was smaller than the pore size of the positive charge yarn mesh, once again demonstrating the superior adhesion of the cotton and jute.

The physicochemical features of the carrier such as patterns and texture (surface roughness) due to the enhancement of cell attachment area play a critical role in the algal attachment (Cui et al. 2013; Schnurr and Allen 2015). Different surface topologies, for example, irregular surfaces with brush-like patterns in cotton and texture in jute carrier in this study, not only increase surface area attachment and allow more microalgae cells to anchor to the solid surface but also reduce the detrimental effects of shear stress and cell detachment (Schnurr and Allen 2015). Christenson et al. also observed a more significant attachment to cotton than to polypropylene, nylon, acrylic, and jute carrier materials (Christenson and Sims 2012). Algal cells attaching to the surface of the carrier material help to receive nutrients and light to the cells, whereas light limitation is a significant issue in suspended systems (Johnson and Wen 2010).

In previous studies, a harvesting strategy for minimizing the cost of microalgae/ cyanobacteria harvesting has been suggested (Borowitzka 1997; Griffiths and Harrison 2009; Bruno et al. 2012).

Attached algae culture systems applying the scraping method are easier to harvest and less expensive than suspended microalgae cultures, which typically use centrifugation as their harvesting process (Lee et al. 2014). In this study, the scraping of the total biomass was performed, with a period of approximately 7–10 days between scrapings. Some literature has noted that much longer scraping intervals disrupted the distribution of light and nutrients in cell algae in the lower biofilm layers. The once-weekly frequency of scraping was more proper for the harvesting of adhered biomass in previous biofilm studies (Fanesi et al. 2019).

### Evaluation of material surface physico-chemical properties on algal attachment

#### Contact angles and surface properties

The negative surface energy of hydrophobic jute and cotton surfaces coated with algae, as shown in Table 1, indicates that solid-solid contact (material surface and algae) is stronger than solid-water interaction. It agrees well with the hydrophobicity results determined by the water contact angle criterion. As expected, the contact angles of low-energy hydrophobic polymers like cotton and jute are usually larger than those of high-energy, more hydrophilic materials like positively charged yarns.

Contrary to our results, which showed a relationship between microalgae attachment and surface energy and contact angle Gross reported poor correlations between cell attachment and the surface energy and water contact angle (Gross 2015). The reason may be due to the different material types, culture conditions, and algal species used in our study.

In this study, the hydrophobicity of the surface after attaching the filamentous cyanobacterium cells to the surfaces could be attributed to the natural hydrophobicity of the cyanobacterium cells. Our result was in agreement with Hao et al. study, which also identified algae *Chlorella vulgaris* as planktonic microalgae and *Anabaena vasriabilis* as a filamentous cyanobacterium, which naturally exhibit hydrophilic and hydrophobic properties, respectively (Hao et al. 2017). In addition, it has been reported that while hydrophobic strains of cyanobacterium *Microsystis* grow into colonies, hydrophilic strains are unicellular, showing increased cell-to-cell attraction with an increase in the hydrophobicity of the cell surface (Yang et al. 2011). Therefore, the observation that microalgae/ cyanobacteria have a hydrophobic surface suggests that hydrophobicity may be one of the main mechanisms promoting the initial adhesion of algal cells (Ozkan and Berberoglu 2013b).

#### Characterization by ATR-FTIR spectroscopy

Cyanobacterium and carriers (positive charge yarn, jute, and cotton) display broad and intense absorption bands at 3000–3500 cm^− 1^corresponding to O-H and N-H stretching vibrations, suggesting that the supports interact with *Desmonostoc* sp. cells (cell-support) through hydrogen bonding. The water intake was shown to have a considerable influence on the CH stretching bands at 2935 and 2900 cm^− 1^ (Célino et al. 2014).

The C-O and C-O-C vibrations associated with polysaccharides on crude jute and jute were covered by the characteristic bands related to *Desmonostoc* sp. at 1036 cm^− 1^ and 1024 cm^− 1^, respectively. The C-O-C band attributed to carbohydrates in raw cotton and cotton was covered by vibrations of *Desmonostoc* sp. at 1174–1134 cm^− 1^, respectively (Duygu et al. 2012). The interaction between polysaccharides on the carrier surface and cells might influence the cell’s initial adhesion to jute and cotton fibres (Li 2022). The vibrations ascribed to carboxylate ions (1400–1398 cm^− 1^) were observable in the spectra of almost all the samples. In all samples, the bands at 1423–1357 cm^− 1^ can be contributed to CH_2_ bending vibrations of methyl (Cheah and Chan 2022). In addition, the absorption band at 2928 cm^− 1^ can be assigned to C-H **(***Desmonostoc* sp.**)** and vibrations at about 2626 cm^− 1^ (raw jute and raw yarn) can be attributed to C = O of amide I and N-H stretch of amide-II, respectively(Duygu et al. 2012). The bands at 1585 and 1481 cm^− 1^ related to N-H bending and amide II C-N stretching vibrations of raw jute and jute were covered by cyanobacterium vibrations. The bands at about 1114 cm^− 1^ attributed to C-O-P and P-O-P in raw cotton and cotton were covered by cyanobacterium vibrations (Cheah and Chan 2022). In general, the results demonstrate that the interaction between jute, yarn, cotton and *Desmonostoc sp.* cells is independent of the surface’s chemical characteristics, and the interaction between the microalgae cells and the surfaces under investigation appears to be physical. The fact that after 90 days of exposure to cyanobacterium, the absorption spectra of jute, cotton, and the yarn did not change, and that none of the functional groups such as sulfate (-SO_4_), amino (—NH_2_), carboxyl (—COOH), hydroxyl (—OH), sulphydryl (—SH), phosphoryl (—PO_3_O_2_), etc. in microalgae were involved in the surface binding process, suggests that the biological activities associated with functional groups of biomass biofilm are preserved.

#### Biomass production and carbohydrate content of attached and suspended systems

In this study compared to the suspended culture system, the attached culture systems (cotton and jute) achieved 3- and 4-times higher biomass productivity. These results are in agreement with Johnson and Wen’s findings that the attached algae culture system produced more biomass than the suspended system (25.65 g DW m^− 2^ and 1.27 ± 0.12 g DW L^− 1^ for the attached and suspended culture biomass yield, respectively) (Johnson and Wen 2010). Several features of algal production were evaluated between the attached and suspended systems. The biomass collected from the attached culture system was paste-like and had a similar water content to the cell pellet centrifuged from the suspension culture system (Johnson and Wen 2010). This means that the attached algal growth system has a significant advantage in terms of biomass harvesting. During suspended development, a substantial amount of water must be evacuated from the algal cells. In addition to making harvesting easier, the attached algal growth system produced more biomass than the suspended system when compared to the lowest amount of biomass production yield in the biofilm system obtained in the yarn carrier (Table 2). The simulated model of suspension and biofilm culture employed in this work revealed that open culture consumes more water than biofilm culture (data not shown). Many studies confirm the fact that a key limiting factor for algal development in terms of economic feasibility and ecological sustainability is the fact that large-scale algae production in suspension systems requires a high amount of fresh water. This constraint is overcome by a considerable reduction in the total volume of culture in the biofilm system (Ozkan et al. 2012; Podola et al. 2016).

Among reports on immobilized-based microalgal systems, biomass production in jute support in this study was consistent with the result described by Hodges et al., who found 4.4 g m^− 2^ day^− 1^ biomass productivity for filamentous cyanobacterium in the RABR system for petroleum wastewater treatment (Hodges et al. 2017). In another study, Gross et al. used a revolving algal biofilm (RBC) system as immobilized cultivation for *Chlorella vulgaris* (UTEX #265) growth and obtained biomass productivity of 4.2 g m^− 2^ day^− 1^ on nylon and polypropylene, which was similar to the productivity observed for jute in this study (Gross et al. 2016). In addition, cotton was also investigated by Christenson and Sims as a substrate in a bench-scale rotating bioreactor of mixed culture (algal-bacterial) and 2.5 g m^− 2^ day^− 1^ productivity was recorded (Christenson and Sims 2012), while in this study, the productivity of 3.6 g m^− 2^ day^− 1^on cotton was obtained. Also, Murphy and Berberoglu reported productivities of 2.8 g m^− 2^ .day^− 1^ for *Anabaena variabilis* as filamentous cyanobacterium in porous substrate bioreactor (PSBR) cultivation (Murphy and Berberoglu 2014). The *Rhizoclonium hieroglyphicum* filamentous green alga was successfully grown on raw and digested dairy effluents in the attachment-based algal turf scrubber (ATS) system, resulting in biomass production of 5.3 g DW m^− 2^ day^− 1^ and 4.9 g DW m^− 2^ day^− 1^ respectively (Mulbry et al. 2008). For *Nitzschia palea* and *Scenedesmus obliquus*, biomass productivity in the algal biofilm culture system was determined by Schnurr et al. at 2.8 and 2.1 g m ^− 2^ d ^− 1^ during the nutrient shortage, respectively (Schnurr et al. 2013).

Microalgae biofilm formed on cotton showed the highest sugar content, followed by jute. In two-way communication, microbial strains, growth phase, external condition, and supporting material are affected by EPS production and their composition. On the other hand, the amount of polysaccharide produced by microbial strains plays a vital role in biofilm formation on a substrate (Sheng et al. 2010; Palma et al. 2017; Jin et al. 2018). As depicted in Table 2, the present study was in line with the previous study’s beliefs that suggested significant relationships between capsular polysaccharides and biomass in microalgae phototrophic biofilm (Bellinger et al. 2005; Pippo et al. 2009).

Two stages are suggested for microorganism adhesion. The first step is reversible attachment, which occurs by macroscopic surface properties, while the second step is irreversible attachment caused by microscopic molecular interaction such as EPS production (Busscher and Weerkamp 1987). It appears that microorganisms with weak initial adhesion to the substrate do not secrete additional EPS. Various amounts of EPS are generated on different surfaces (Becker 1996). In addition to their adhesion and cell-protective roles, EPS have other functions including nutrient trapping, detoxification and microcolony formation (Becker 1996). As a result, cotton and jute proved to be more effective at capturing nutrients, creating an initial microcolony, and developing biofilm with a higher EPS content during the course of this research. It is also important to note that physicochemical properties, microalgae EPS secretion, and the reduction of the free energy in a flowing system, as well as the decrease in interfacial tension between surface and cell attachment, are all influencing biological factors that alter surface characteristics and minimize free energy in a flowing system. It is also important to note that physicochemical properties, microalgae and cyanobacterium EPS secretion, and the reduction of the free energy in a flowing system, as well as the decrease in interfacial tension between surface and cell attachment, are all influencing biological factors (Simões et al. 2008; Barros et al. 2018). Thus, high EPS content increases the hydrophobic interaction and cell adhesion to surfaces (Moghaddam et al. 2018).

Biofilms can be strengthened by rougher surfaces, such as those found in jute and cotton, which increase the production of EPS and bind cells together (Schnurr and Allen 2015). Compared to our previous studies, HPLC data (data not shown) confirmed that there was no difference in the monosaccharide composition of EPS, which was extracted from single-cell and filamentous strains and was composed mainly of structural units of glucose, glucosamine and sucrose (Mousavian et al. 2022).

The widespread application of microalgal biofilms in wastewater treatment, biomass and high-value metabolites production is prevalent today. Treatment of wastewater is a significant application of microalgal biofilm following biodiesel generation (Miranda et al. 2017). It would be ideal to use microalgal biofilm biomass as fertilizer if wastewater did not contain heavy metals (Patwardhan et al. 2022).

In general, the formation of microalgal biofilms seems to be species-dependent and affected by physico-chemical surface features. This study examined a rotating biofilm system for algal culture using different supporting materials. Filamentous strain cyanobacterium and jute and cotton have been identified as appropriate algae strains and effective supporting materials, respectively, for the establishment of biofilm algal culture. In comparison to the suspended growth culture operated under similar conditions, the biofilm system achieved greater biomass production. In this study, the biomass productivity of the rotating cylinders for jute and cotton was 4.76 and 3.61 g m^− 2^ day ^− 1^, and the biomass production was 66.74 and 44.41 g m^− 2^, respectively. The production of microalgal and cyanobacterial biomass can be facilitated by biofilm-based microalgal systems. This approach applies to a wide variety of low-cost and long-lasting supporting materials, which has attracted more attention from researchers and industrialists.

## Data Availability

The data that support the findings of this study are available from the corresponding author upon reasonable request.
